# Electron microscopy reveals novel external specialized organs housing bacteria in eagle ray tapeworms

**DOI:** 10.1371/journal.pone.0244586

**Published:** 2021-01-22

**Authors:** Janine N. Caira, Kirsten Jensen

**Affiliations:** 1 Department of Ecology & Evolutionary Biology, University of Connecticut, Storrs, Connecticut, United States of America; 2 Department of Ecology & Evolutionary Biology and the Biodiversity Institute, University of Kansas, Lawrence, Kansas, United States of America; CNRS: BIOM Integrative Biology of Marine Organisms, FRANCE

## Abstract

Nutritionally-based mutualisms with bacteria are known to occur in a wide array of invertebrate phyla, although less commonly in the Platyhelminthes. Here we report what appears to be a novel example of this type of association in two geographically disparate and phylogenetically distant species of tapeworms of eagle rays—the lecanicephalidean *Elicilacunosus dharmadii* off the island of Borneo and the tetraphyllidean *Caulobothrium multispelaeum* off Senegal. Scanning and transmission electron microscopy revealed that the grooves and apertures on the outer surfaces of both tapeworms open into expansive cavities housing concentrations of bacteria. This led us to reject the original hypothesis that these structures, and their associated mucopolysaccharides, aid in attachment to the host mucosa. The cavities were found to be specialized in-foldings of the tapeworm body that were lined with particularly elongate filitriches. Given tapeworms lack a gut and employ filitriches to assist in nutrient absorption, enhanced nutrient uptake likely occurs in the cavities. Each tapeworm species appeared to host different bacterial monocultures; those in *E*. *dharmadii* were coccoid-like in form, while those in *C*. *multispelaeum* were bacillus-like. The presence of bacteria in a specialized structure of this nature suggests the structure is a symbiotic organ. Tapeworms are fully capable of obtaining their own nutrients, and thus the bacteria likely serve merely to supplement their diet. Given the bacteria were also extracellular, this structure is more consistent with a mycetome than a trophosome. To our knowledge, this is not only the first evidence of an external symbiotic organ of any type in a nutritionally-based mutualism, but also the first description of a mycetome in a group of invertebrates that lacks a digestive system. The factors that might account for the independent evolution of this unique association in these unrelated tapeworms are unclear—especially given that none of their closest relatives exhibit any evidence of the phenomenon.

## Introduction

Symbiotic associations with bacteria are widespread across invertebrate taxa, having been reported from numerous invertebrate phyla [1, 2]. Many of these associations are mutualisms that have a nutritional basis [2–4]. Among nutritionally-based mutualisms, some bacteria are ectosymbionts (also referred to as epibionts) associated with the outer surface of the body [5–7], gills [8–10], mantle cavity [11], or atrial cavity [12] of their invertebrate host. More commonly, the bacteria are endosymbionts (also referred to as endobionts), many of which are associated with the digestive tract [12–15], or excretory organs [3, 14, 16, 17] of their invertebrate host. Yet other endosymbiotic bacteria occupy specialized structures known as symbiotic organs [18], which are generally of two basic types. "Trophosomes" consist of compact masses of bacteriocytes that house intracellular bacteria. These structures are found in invertebrates, or stages of invertebrates, that lack a digestive system. The nutrients captured by the bacteria they house are the primary or sole source of nutrition for the invertebrate host and thus they essentially serve as a functional replacement of the gut [2, 19]. "Mycetomes", sometimes also referred to as "bacteriomes", occur in a variety of configurations. In many insects they consist of solid masses of cells (i.e., bacteriocytes) that house intracellular bacteria [3]. However, in leeches, for example, they also occur as tubes or sacs, the lumen of which houses bacteria [14]. In general, mycetomes are found in invertebrates that possess a gut. The bacteria in these structures supplement the diet of their invertebrate hosts by providing nutrients their hosts are unable to synthesize themselves [4].

Although relatively rare, nutritionally-based associations with bacteria are known to occur in some members of the phylum Platyhelminthes. Marine catenulids of the genus *Paracatenula*, all of which lack a digestive system, live in association with dense concentrations of chemosynthetic bacteria that occupy bacteriocytes in a trophosome that extends throughout much of the length of their body [20, 21]. Bacteria have also been reported intimately associated with the external surfaces of several groups of parasitic platyhelminths including digeneans [22, 23] and a diversity of freshwater fish tapeworms [24–27]. Although the exact nature of these associations is uncertain, they were postulated to be nutritionally-based mutualisms by most of these authors.

Bacterial associations with tapeworms are especially interesting because this group of platyhelminths also lacks all elements of a digestive system. As adults, tapeworms live in the digestive system of their vertebrate host absorbing nutrients in the form of small sugars [28], through a specialized syncytial outer layer of their body known as a neodermis. The surface of this outer body layer bears unique extensions called microtriches, which vary in form across tapeworm groups [29]. However, all tapeworms possess the microthrix form known as a filithrix. These long, slender structures serve to increase the worm's absorptive surface area to enhance nutritional uptake [30–33]. In the cases of bacteria associated with freshwater fish tapeworms, individual bacteria have been observed attached to the filitriches or to the surface of the neodermis between filitriches using a variety of structures such as filaments, tufts, etc. [24, 25, 27].

The present study was motivated by the discovery of the second occurrence of a puzzling structure in form of longitudinal grooves and tandem series of openings on the dorsal and ventral external surfaces of the body of a marine, elasmobranch-hosted tapeworm. The original observation was made in three species of the order Lecanicephalidea in the genus *Elicilacunosus* found parasitizing two species of eagle rays off Borneo [34]. The second was in a tapeworm of the order Tetraphyllidea in the genus *Caulobothrium* found parasitizing the duckbill eagle ray off Senegal [35]. In both cases, the lining of the structure was found to stain positively with McManus' periodic acid Schiff (PAS) suggesting the presence of some sort of mucopolysaccharides [36]. This finding led the authors to propose the structure might assist the tapeworm with attachment to the surface of the mucosa of its elasmobranch host. But that function remains to be confirmed.

Uncertainty surrounding the nature and function of this structure led us to explore it in more detail using electron microscopy. Ultrastructural examination indicates it performs an entirely different, and very unexpected, function. In both groups of tapeworms, the grooves and openings were found to expand into a cavity housing concentrations of bacteria. The intimate nature of the associations we observed between the bacteria occupying the cavity and the highly elongate filitriches of the lining of the cavity leads us to believe there may be a nutritional basis to this association. To our knowledge, symbiotic organs for housing bacterial partners in nutritionally-based associations are known only from endosymbiotic associations. However, the structure seen in these tapeworms is an external symbiotic organ. Although gutless, tapeworms are able to absorb nutrients through the outer layer of their body, which suggests these bacterial associates are merely supplementing the diet of their tapeworm. If our interpretation is correct, we believe this is not only the first instance of any type of external symbiotic organ, but it is also the first instance of a mycetome in a group of invertebrates that lacks a digestive system.

## Materials and methods

### Sampling

Specimens of the lecanicephalidean tapeworm species *Elicilacunosus dharmadii* were collected from the spiral intestine of a banded eagle ray (*Aetomylaeus nichofii*) off Mukah, on the island of Borneo, in June 2002. Specimens of the tetraphyllidean tapeworm species *Caulobothrium multispelaeum* were collected from the spiral intestine of the duckbill eagle ray (*Aetomylaeus bovinus*) from the eastern Atlantic Ocean off Diogue, Senegal, in January 2005. Specimens were fixed in seawater-buffered formalin (9:1) and transferred to 70% ethanol after several weeks for storage. All collections were conducted following the University of Connecticut Institutional Animal Care and Use protocols No. C0100101 and C0100102 (for June, 2002) and A04-177 (for January, 2005).

### Morphology

#### Scanning electron microscopy (SEM)

A complete worm of *E*. *dharmadii*, and five complete worms and one free proglottid of *C*. *multispelaeum* were prepared for and examined with SEM as follows. They were dehydrated in a graded ethanol series, transferred to a solution of 1% osmium tetroxide overnight, hydrated in a graded ethanol series, transferred to hexamethyldisilazane, and allowed to air dry in a fume hood for 1 hr. They were mounted on aluminum stubs on double-sided PELCO carbon tabs, sputter coated with 35 nm of gold/palladium, and examined with an FEI Versa 3D Dual Beam (FEI Company, Hillsboro, OR) or an FEI Nova NanoSEM 450 (FEI Company) field emission scanning electron microscope.

#### Transmission electron microscopy (TEM)

One free proglottid of *E*. *dharmadii* and two free proglottids of *C*. *multispelaeum*, both originally fixed in seawater-buffered formalin, were prepared and examined with TEM as follows. Specimens were re-dehydrated in a graded ethanol series and post-fixed at 4° C (pH 7.3) for 3.5 hr in a solution of 1.5% glutaraldehyde, 1.5% paraformaldehyde, 0.1 M HEPES buffer, 0.08 M NaCl, and 3 mM MgCl_2_. Following three rinses in buffer, specimens were secondarily fixed in a solution of 1% osmium tetroxide, 0.8% potassium ferricyanide, 0.08 M NaCl, and 3 mM MgCl_2_ in 0.1 M HEPES buffer (pH 7.3) in the dark for 1.5 hours. After two rinses in cold purified water, specimens were dehydrated in a graded ethanol series and cleared by transfer to propylene oxide twice for 10 min each. Specimens were infiltrated in an epoxy resin composed of Embed-812, Araldite GY 506, and dodecenyl succinic anhydride. Initial infiltration was conducted in a 1:1 mixture of resin:propylene oxide for 2 hours followed by a 3:1 mixture of resin: propylene oxide overnight. The following day infiltration was completed by adding of 2,4,6-Tri-Dimethylamino-ethylphenol to the resin and the specimens were incubated for 6 hours with one change of resin after 3 hours. Specimens were oriented for cross-sectioning and embedded in flat double end molds (Ted Pella, Inc.). Ultrathin sections were taken with an ultra 45° Diatome™ diamond knife on a Leica Ultracut UCT microtome (Leica, Bannockburn, IL) and collected on slot grids (Ted Pella, Inc.). Grids were stained in 2% uranyl acetate and 2.5% Sato**’** s lead citrate, washed with water, and dried at room temperature. Images were obtained using an FEI Tecnai BioTwin G2 Spirit transmission electron microscope (FEI Company) equipped with an AMT 2k XR40 CCD (4 megapixel) camera (Advanced Microscopy Techniques Corp., Danvers, MA) at an accelerating voltage of 80 kV.

## Results

### Elicilacunosus dharmadii

**Fig 1:**
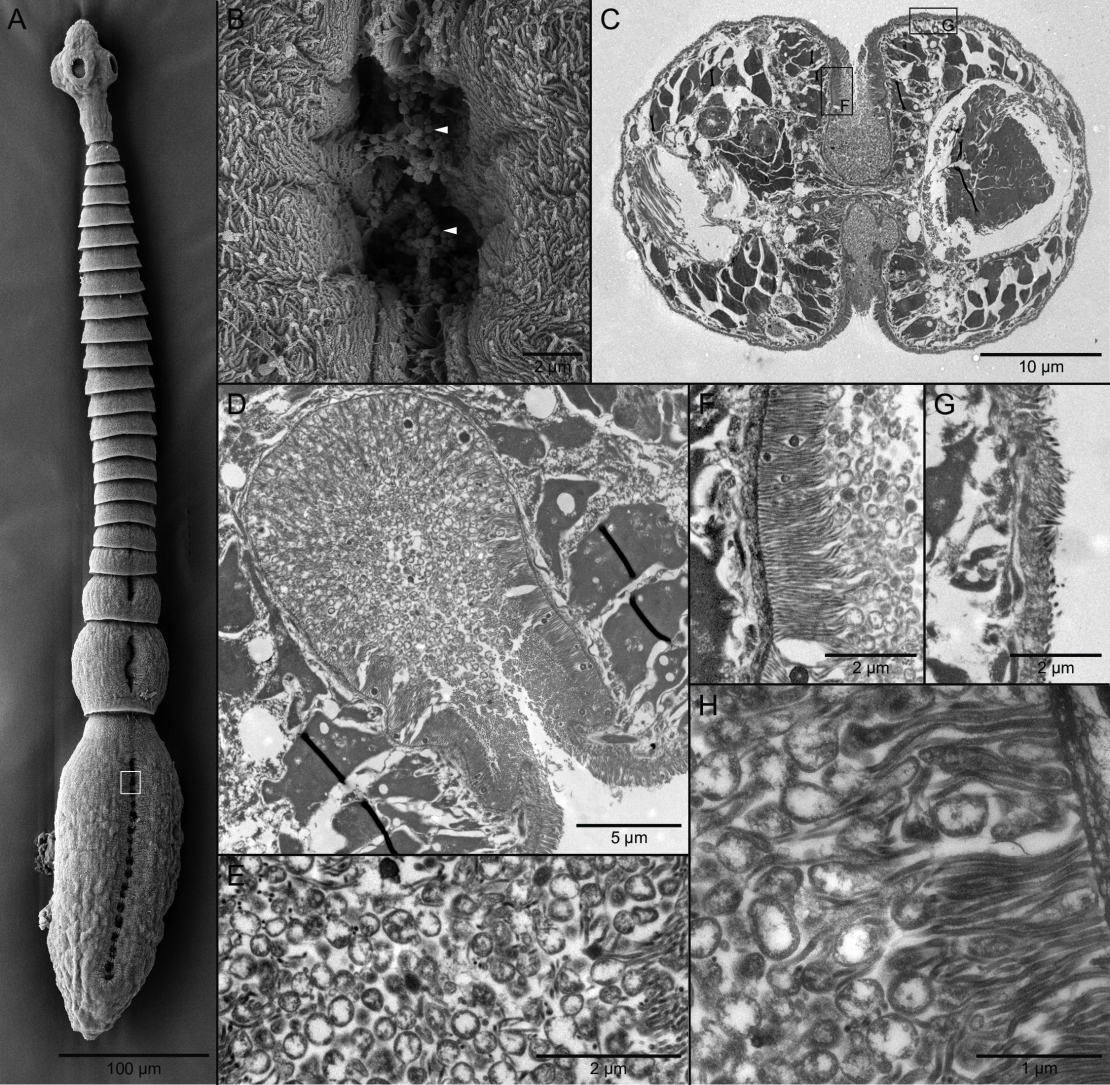
Scanning (SEM) and transmission (TEM) electron micrographs of *Elicilacunosis dharmadii* (Cestoda: Lecanicephalidea). (A) SEM of whole worm showing tandem series of apertures on terminal mature proglottid; white rectangle indicates location of detail in B. (B) SEM of apertures of symbiotic organ with coccoid-like bacteria (white arrowheads). (C) TEM of cross section through symbiotic organs on dorsal and ventral surfaces of mature proglottid; rectangles indicate locations of F and G. (D) TEM of cavity of symbiotic organ with dense concentrations of bacteria. (E) TEM of bacteria in cavity. (F) TEM of elongate filitriches lining surface of cavity of symbiotic organ. (G) TEM of shorter filitriches on other body surfaces. (H) TEM of bacteria nestled among elongate filitriches lining cavity of symbiotic organ.

Scanning electron microscopy showed grooves on the dorsal and ventral surfaces of the posterior-most immature proglottids; these transformed into a tandem series of apertures on the dorsal and ventral surfaces of the terminal mature proglottid (Fig 1A). Clusters of coccoid-like (i.e., spherical) bacteria were visible in the apertures of the mature proglottid (Fig 1B). Elongate filitriches were seen on the surfaces of the apertures (Fig 1B).

Transmission electron microscopy of a cross section through a mature proglottid revealed that the apertures on the dorsal and ventral surfaces of the body open into expansive cavities hat extend to the center of the body of the worm (Fig 1C). The cavities appear to represent in-foldings of the dorsal and ventral external surfaces. Dense concentrations of bacteria were visible within the cavities (Fig 1D). For the most part, these bacteria were similar in form; they were coccoid-like in shape (Fig 1E) and approximately 300–450 nm in diameter. The dense elongate filitriches extending from the surface of the lining the cavities (Fig 1F) were found to be considerably longer (i.e., 1.89–2.1 μm in length) than those of the body surfaces outside of the grooves (i.e., 0.66–0.77 μm in length) (Fig 1G). Numerous bacteria were seen nestled among the elongate filitriches lining the cavities of the dorsal and ventral surfaces (Fig 1H). In combination, the SEM and TEM observations lead us to interpret these structures as symbiotic organs.

### Caulobothrium multispelaeum

**Fig 2:**
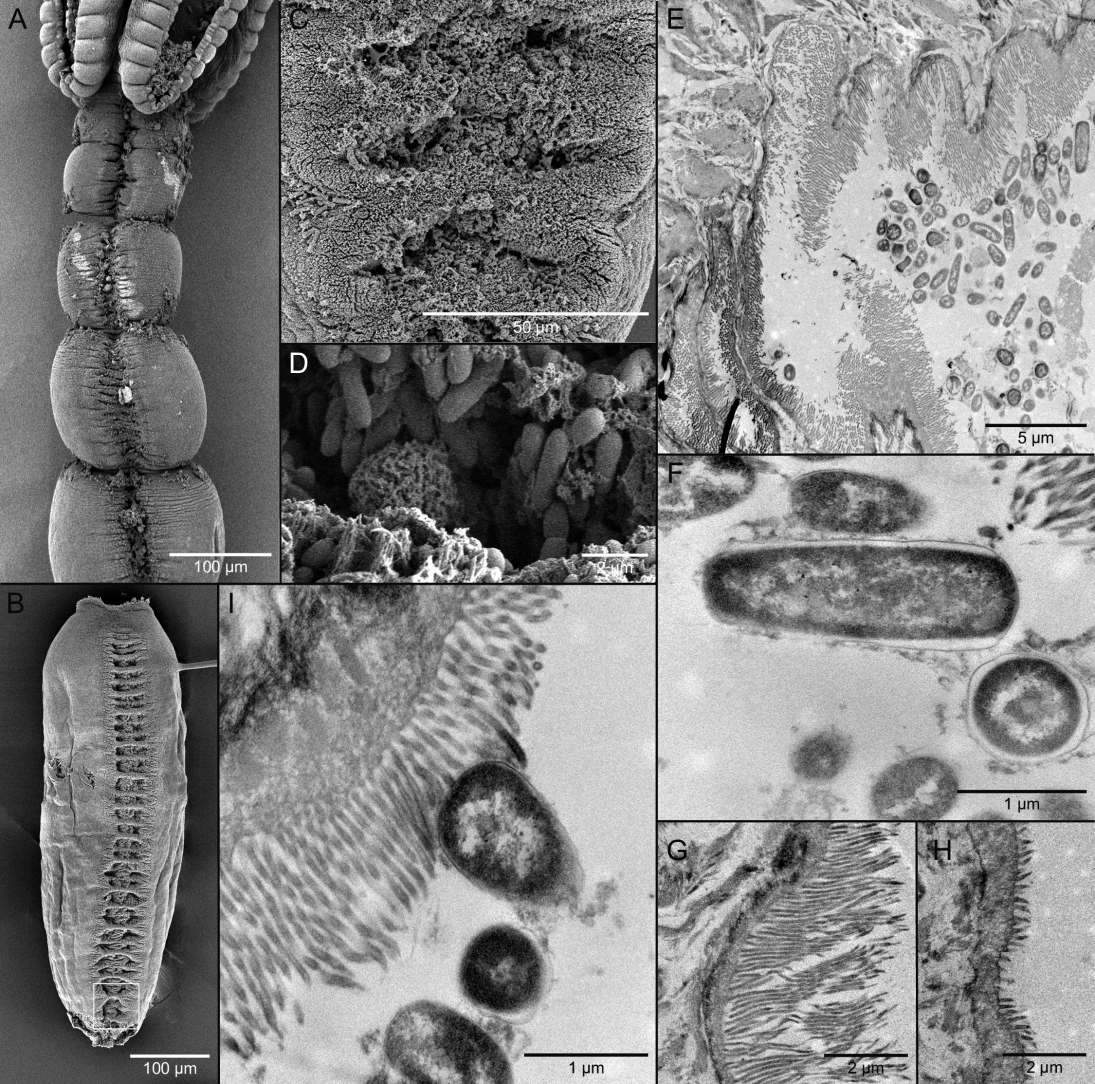
Scanning (SEM) and transmission (TEM) electron micrographs of *Caulobothrium multispelaeum* (Cestoda: Tetraphyllidea). (A) SEM of anterior portion of worm showing grooves of symbiotic organ in immature proglottids. (B) SEM of mature proglottid showing tandem series of elliptical apertures of symbiotic organ; white rectangle indicates location of detail in C. (C) SEM of apertures of symbiotic organ. (D) SEM of bacillus-like bacteria in apertures of symbiotic organ. (E) TEM of cross section through portion of symbiotic organ showing bacteria in cavity. (F) TEM of bacteria in cavity in frontal and cross section. (G) TEM of elongate filitriches lining surface of cavity of symbiotic organ. (H) TEM of shorter filitriches on other body surfaces. (I) TEM of bacterium in intimate contact with filitriches lining cavity of symbiotic organ.

Scanning electron microscopy showed grooves on the immature proglottids throughout the length of the strobila (Fig 2A). These developed into a tandem series of elliptical apertures on the dorsal and ventral surfaces of the mature proglottids (Fig 2B). Aggregations of bacteria were visible in the apertures and the surfaces immediately adjacent to the apertures of the mature proglottids (Fig 2C). These bacteria were bacillus-like (i.e., rod-shaped) in form (Fig 2D). Elongated filitriches were visible surrounding the bacteria in the apertures (Fig 2C).

Owing to our use of slot grids, we were unable to obtain an image of a complete cross section through a mature proglottid of *C*. *multispelaeum* with TEM. However, images of partial sections of a mature proglottid indicated that the apertures on the dorsal and ventral surfaces also opened into expansive cavities, which appear to represent in-foldings of the external surfaces of the worm (Fig 2E). Numerous bacteria were visible in the cavities (Fig 2E). The bacteria were generally bacillus-like in form and 2–3 μm long by 1–1.5 μm wide in cross section (Fig 2F). Dense concentrations of elongate filitriches were seen extending from the surface of the lining of the cavities (Fig 2G). At 3.6–4.1 μm in length, these microtriches were much longer than those on the other surfaces of the body, which were 1.1–1.3 μm in length (Fig 2H). A number of bacteria were observed nestled among the highly elongate filitriches lining the cavities (Fig 2I) of what we are also interpreting in this species as symbiotic organs.

## Discussion

Several lines of evidence indicate that the association between these elasmobranch-hosted tapeworms and the bacteria that occupy their external cavities is a nutritionally-based mutualism. The role filitriches play in assisting with the absorption of nutrients is supported by an extensive body of literature [32]. The fact that the filitriches on the surfaces of the lining of the cavities of these tapeworms were much longer than those on the remainder of the body, suggests that the filitriches in the cavity serve to enhance the uptake of nutrients in this region of the body. In both cases, bacteria were observed in intimate, direct contact with filitriches of the lining of the cavities. It has been suggested that, given the end-products of tapeworm carbohydrate catabolism are molecules such as lactate, succinate, and acetate, it seems plausible that, by attaching to the surface of a tapeworm, bacteria are able to use these organic acids as a source of carbon [24]. In return, it was postulated that the associated bacteria produce enzymes capable of hydrolyzing complex molecules such as carbohydrates and proteins, thereby making these products available to their gutless tapeworm hosts for assimilation using membrane digestion and active transport [24]. It has similarly suggested that enzymes produced by tapeworm-associated bacteria play a role in assisting with tapeworm digestion [25]. It seems reasonable to propose that similar metabolic exchanges could be occurring in the specialized external cavities of these elasmobranch-hosted tapeworms. The cavities also appear to represent a stable, protected environment that serves to physically isolate the bacteria from the environment of the elasmobranch gut. The fact that no sign of pathology at the site of bacterial contact with the tapeworm host was observed with SEM or TEM here, or previously in freshwater tapeworm systems [24–27, 37, 38], lends further support to the hypothesis that these are mutualistic associations.

What makes the associations examined here unique relative to those seen in freshwater fish tapeworms is that, rather than individual bacterial symbionts using structures of their own making (tufts, filaments, etc.) to attach to the surface of their freshwater fish tapeworm hosts [24–27], the bacteria observed here exhibited no modifications of their own. Instead, the tapeworms with which the bacteria were associated exhibited specialized symbiotic organ in the form of in-foldings of the dorsal and ventral surfaces of their body that accommodate their bacterial symbionts. The freshwater fish tapeworms exhibited no such structures.

Several factors lead us to believe the concentrations of bacteria seen associated with the cavities is not merely an artifact of the bacteria on the more accessible surfaces of the tapeworm body having been removed as a result of processing. Some bacteria were observed protruding from and even spilling out of the apertures onto the adjacent surfaces of the tapeworm body with SEM (Figs 1B, 2C). Their presence on such exposed surfaces argues against bacteria being easily removed from all but less accessible portions of the body over the course of processing. In addition, the bodies of both groups of tapeworms offer a variety of other "protected" regions beyond the cavities, including folds of the scolex and overlapping margins of adjacent proglottids, none of which were observed to house bacteria. Finally, if the bacteria in the cavities represented merely a portion of the normal microbiome of the elasmobranch gut that failed to be removed by processing, one would expect the bacteria in the cavities to be highly heterogeneous and this is clearly not the case in either tapeworm taxon.

The reasons for characterizing this structure as a symbiotic organ are threefold. First, it clearly represents a specialized structure. Not only are the dorsal and ventral surfaces of the body of the tapeworm invaginated to form cavities that open to the outside in younger proglottids via a longitudinal groove and in older proglottids as a tandem series of openings, but the filitriches lining the cavities are conspicuously longer than those on all other surfaces of the body. Second, the ubiquitous presence of the structure in both groups of tapeworms suggests the association is a normal element of the life of these parasites; the structure was found in all specimens of each of the three species of *Elicilacunosus* [34] and also in all specimens of *C*. *multispelaeum* examined [35]—a total of 72 worms—in addition to those examined here. Third, the fact that the structure occurs in proglottids of various ages (i.e., immature and mature proglottids) indicates this is a stable, long-term association with the adult worm. The structure also occupies a substantial portion of the body of the tapeworm. Whether this is an obligate association remains to be determined, but it fulfills all of the criteria typically used to describe a symbiotic organ [1, 4, 18].

With respect to the type of symbiotic organ, we consider it to be a mycetome rather than a trophosome for the following reasons. Although tapeworms lack a digestive system, their ability to effectively obtain nutrients through their syncytial outer neodermis using a variety of mechanisms including membrane digestion, active transport, etc. is well documented [28, 32]. There is no evidence that species of *Elicilacunosus* or *C*. *multispelaeum* lack this ability; the ultrastructure of the neodermis in both species examined was fully intact. This suggests the bacterial associates are likely serving merely to supplement the diets of these tapeworms. Finally, unlike bacteria housed in trophosomes, which are entirely intracellular, the bacteria housed in the structure in elasmobranch-hosted tapeworms are extracellular. To our knowledge, this therefore represents the first example of an invertebrate with a nutritionally-based association with bacteria in which the specialized structure housing the bacteria is located on the outside of the body of the invertebrate host. Furthermore, we believe this is the first instance of a mycetome that houses ectosymbiotic, rather than endosymbiotic, associates.

Several different types of bacteria have been reported from the surfaces of freshwater fish tapeworms. These range from nanobacteria [26, 27, 37, 38], to larger rod-like, cocci-like, and disc-like and uncommon forms [24–26]. It was beyond the scope of this project to fully characterize the bacteria that inhabit the cavities of either tapeworm species. However, our results indicate that the bacterial floras associated with both tapeworm groups are highly homogeneous and may represent monocultures. Our results further indicate that the monocultures hosted by the two tapeworm groups differ. In *E*. *dharmadii* the bacteria are coccoid-like in form; in *C*. *multispelaeum* they are bacillus-like in form. Unfortunately, we were not able to expand on the identification of the bacteria further. Our attempt to extract bacterial DNA from the single proglottid of *C*. *multispelaeum* fixed in ethanol available to us for study failed and thus we were not able to advance the identities of these bacteria further using molecular tools. The fact that our specimens were originally fixed for light, rather than electron, microscopy also limits identification based on ultrastructural details. A viable explanation for why the two groups of tapeworms host different monocultures of bacteria is not immediately apparent. Definitive identifications of the bacteria involved may provide clues to help inform this question.

Our results cause us to re-evaluate the significance of the fact that the linings of these cavities were found to stain positively with PAS [34, 35]. Given that the bacterial associates occupy what is essentially an external site on the tapeworms and the tapeworms live in the digestive system of their elasmobranch hosts, the mode of transmission of the bacterial associates to the tapeworms seems more likely to be horizontal, than vertical. In which case, the rich bacterial flora of the gut of the elasmobranch hosting the tapeworms would be the best candidate as the environmental source of the bacterial symbionts. The importance of host-secreted mucus in horizontal transfer, not only in establishing initial physical encounters between symbiont and host partners, but also in the role they play as the final residence for the symbiont is clear [39]. This leads us to propose that, rather than aiding in attachment to the host mucosa as originally hypothesized [34, 35], the mucopolysaccharides produced by the lining of the cavities of *E*. *dharmadii* and *C*. *multispelaeum* play a key role in transmission by establishing and maintaining associations with bacteria.

One of the most intriguing aspects of these findings is identifying a viable scenario to explain the independent evolution of a unique relatively elaborate strategy for dietary supplementation in two geographically distant and phylogenetically unrelated species of tapeworms. In this context it is interesting to examine commonalities between *Elicilacunosus* and *C*. *multispelaeum*. At 0.54–3.4 mm in total length, all four are some of the smallest species of elasmobranch tapeworms known. All four species also parasitize myliobatiform stingrays of the genus *Aetomylaeus*. Yet, neither of these features is unique to these four species. For example, adults of the elasmobranch-hosted lecanicephalideans *Quadcuspibothrium francisi*, *Healyum harenamica*, and *Healyum pulvis* are all less than 1 mm in total length. Furthermore, all three species of *Aetomylaeus* that host *Elicilacunosus* or *C*. *multispelaeum* are parasitized by numerous other tapeworm species, none of which exhibit this type of specialized structure [34, 35]. We remain at a loss to explain why these two taxa alone, among the more than 1,000 other species of tapeworms that parasitize elasmobranchs [40], exhibit this association.

The traditional notion that nutritional symbionts share long evolutionary histories with their animal hosts [41] does not seem to apply here. While all three species of *Elicilacunosus* engage in this symbiotic relationship, that is not the case for their sister genus *Collicocephalus* [42] or any other member of the order Lecanicephalidea. Nor is *Elicilacunosus* an especially early diverging branch on the lecanicephalidean tree resulting from molecular phylogenetic analyses [42]. Of the ten members of its genus, *Caulobothrium multispelaeum* is the only species that exhibits this association. This is especially interesting because its sister species, *C*. *katzi* [43], also parasitizes *A*. *bovinus* [23, 35]. It is hard to believe that the metabolic capabilities of the species that engage in these symbiotic associations differ substantially from those of their closest relatives that do not. However, it has been noted that specialized organs are often absent from close relatives that do not engage in such associations, largely because the organs develop in response to colonization by compatible microorganismal partners [18].

Clearly, this system requires more extensive investigation. The taxonomic identity of the associated bacteria would be interesting to determine and might provide further insight into the hypothesized nutritional basis of this relationship, which would be even further advanced with proteomic work. Comparative genomic and transcriptomic work would be highly enlightening from the standpoint of the evolution of this phenomenon in this unlikely pair of genera. Characterization of the bacterial floras of the spiral intestines of the host stingrays could expand our understanding of the mode of transmission and the source of the bacterial symbionts. Biochemical characterization of the mucopolysaccharides associated with the linings of the cavities of the mycetomes would help establish whether these compounds play a role in transmission or maintaining the association. We have reported this association in adult tapeworms; investigation as to whether this association is also found in the trophically-transmitted larval stages of these species would provide insight into the longevity of the association and mode of transmission. Unfortunately, any future research on this system will require the collection of new material of these tiny tapeworms. This will be particularly challenging given they are known only from eagle rays off Borneo and Senegal.

## Conclusions

Scanning and transmission electron microscopy confirm that the unique specialized cavities found on the dorsal and ventral external surfaces of the body of two distantly related groups of tapeworms of stingrays house dense concentrations of bacteria. The intimate nature of the association between these bacteria and the finger-like surface elaborations (i.e., filitriches) lining these cavities, which are typically considered to aid with the absorption of nutrients, suggests there is a nutritional basis to this association. Although tapeworms lack all elements of a digestive system, they readily absorb nutrients from the digestive system of the vertebrate hosts in which they reside through their outer neodermis and thus do not rely solely on this association with bacteria for nutrient acquisition. As a consequence, these symbiotic organs do not fully conform to the traditional definitions of either a trophosome or a mycetome. Given tapeworms are able to obtain some nutrients on their own, they are considered to be the first instances of external mycetomes.
